# Integrative genomic, proteomic and phenotypic studies of *Leishmania donovani* strains revealed genetic features associated with virulence and antimony-resistance

**DOI:** 10.1186/s13071-020-04397-4

**Published:** 2020-10-12

**Authors:** Zhiwan Zheng, Jianping Chen, Guangxu Ma, Abhay R. Satoskar, Jiao Li

**Affiliations:** 1 Department of Pathogenic Biology, West China School of Basic Medical Sciences and Forensic Medicine, Sichuan University, Chengdu, China; 2 Animal Disease Prevention and Food Safety Key Laboratory of Sichuan Province, Chengdu, China; 3 College of Animal Sciences, Zhejiang Provincial Key Laboratory of Preventive Veterinary Medicine, Zhejiang University, Hangzhou, China; 4 Department of Veterinary Biosciences, Melbourne Veterinary School, The University of Melbourne, Parkville, Victoria Australia; 5 Department of Pathology, Ohio State University Medical Center, Ohio State University, Columbus, USA; 6 Department of Microbiology, Ohio State University, Columbus, USA

**Keywords:** *Leishmania donovani*, Antimony resistance, Whole genome resequencing, Proteome profiling, Genetic variations

## Abstract

**Background:**

Leishmaniasis is a neglected tropical disease affecting millions of people worldwide. Emerging drug resistance of *Leishmania* species poses threaten to the effective control and elimination programme of this neglected tropical disease.

**Methods:**

In this work, we conducted drug-resistance testing, whole genome resequencing and proteome profiling for a recently reported clinical isolate with supposed drug resistance (HCZ), and two reference sensitive strains (DD8 and 9044) of *Leishmania donovani*, to explore molecular mechanisms underlying drug resistance in this parasite.

**Results:**

With reference to DD8 and 9044 strains, HCZ isolate showed higher-level virulence and clear resistance to antimonials in promastigote culture, infected macrophages and animal experiment. Pairwise genomic comparisons revealed genetic variations (86 copy number variations, 271 frameshift mutations in protein-coding genes and two site mutations in non-coding genes) in HCZ isolate that were absent from the reference sensitive strains. Proteomic analysis indicated different protein expression between HCZ isolate and reference strains, including 69 exclusively detected proteins and 82 consistently down-/upregulated molecules in the HCZ isolate. Integrative analysis showed linkage of 12 genomic variations (gene duplication, insertion and deletion) and their protein expression changes in HCZ isolate, which might be associated with pathogenic and antimony-resistant phenotype. Functional annotation analyses further indicated that molecules involved in nucleotide-binding, fatty acid metabolism, oxidation-reduction and transport might play a role in host-parasite interaction and drug-resistance.

**Conclusions:**

This comprehensive integrative work provided novel insights into the genetic basis underlying virulence and resistance, suggesting new aspects to be investigated for a better intervention against *L. donovani* and associated diseases.
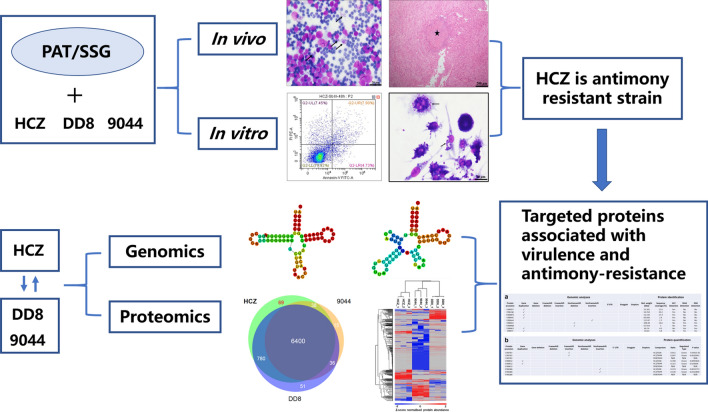

## Background

Leishmaniasis, a neglected tropical disease, is caused by the infection with *Leishmania* species, affecting millions of people in approximately 100 endemic countries [1, 2]. This parasitic disease is transmitted by the bite of phlebotomine sand flies, and have three main forms: cutaneous, mucocutaneous and visceral leishmaniasis (VL) [3]. Although most people have a silent infection, some advanced cases of cutaneous leishmaniasis can cause ulcers, and VL is almost always fatal without proper treatment, especially the advanced one [3, 4]. In the past decades, the intensive vector control and a better access to diagnosis and treatment facilities have successfully and substantially reduced the number of VL in Asia [2, 5–7]. However, leishmaniasis remains to be one of the poverty-related diseases in underprivileged countries and regions [1, 2, 8]. No vaccines are currently available for clinical use to prevent the infection and reinfection by *Leishmania* species, although progress has been made in this field [9–11]. Chemotherapy is still the first-line option for the treatment and elimination of *Leishmania* infection and related diseases [12, 13].

Drug resistance of *Leishmania* species usually results in treatment failure and relapse of leishmaniasis, posing a considerable threaten to the effective control of this disease [14]. Although liposomal amphotericin B, miltefosine and pentavalent antimonials have been widely used for the treatment of VL in the past decades, an increase of treatment failure and sequela of infection has been frequently reported in recent years, particularly in endemic countries and regions (e.g. Nepal and Southeast Asia region) [15–18]. Unfortunately, molecular mechanisms underlying the establishment of drug resistance have not yet been clearly elucidated [19], which significantly hinders the discovery of novel intervention strategies (e.g. new drugs or vaccines), and challenges the elimination of leishmaniasis by 2020 in endemic areas. Tackling these issues and challenges requires a better understanding of the molecular basis underlying drug resistance in *Leishmania* species.

In the past 30 years, progress has been made in unravelling the infection, genetics and evolution of *Leishmania* spp. at the molecular level [20–22]. Decoding the genomes of *Leishmania* species marked an important turning point for the molecular exploration of these pathogens [23–25]. Particularly, recent genomic and transcriptomic studies of *Leishmania* species significantly advanced our understanding of the genetic plasticity and molecular differences between species/strains, which might be associated with the resistance to antileishmanial drugs and treatment failure [22, 26–29]. However, it is still challenging to integrate and interpret the genetic features revealed at DNA and RNA levels to elucidate the genetic basis for drug resistance in protozoan parasites. Clearly, there is a strong need to explore the genetic plasticity and biological variations of *Leishmania* species at genomic and post-genomic (transcriptomic, proteomic and metabolomic) levels [30, 31].

In this study, we conducted antimony-resistant testing, genomic sequencing and proteomic profiling of a clinical isolate (HCZ) and two drug-susceptible (DD8 and 9044) strains of *L. donovani*. Comparative studies were performed to identify genetic variations and specificities at DNA and protein levels between the resistant and susceptible phenotypes. Molecular mechanisms underlying virulence and resistance in *L. donovani* were explored by comparing and integrating the phenotypic features, genomic variations and proteomic differences among HCZ isolate, DD8 and 9044 strains.

## Methods

### Procurement of parasites, drugs, cell and animals

A *Leishmania* clinical isolate (MHOM/CN/2016/SCHCZ) was obtained from bone marrow puncture fluid of a VL patient at West China Hospital, Sichuan University, China. The patient was treated with 4 courses of antimonials treatment and then relapsed. The bone marrow puncture fluid was obtained after the relapse, then inoculated with three laboratory golden hamsters by intraperitoneal injection, 0.3 ml per hamster. Clinical isolate (MHOM/CN/2016/SCHCZ) and reference sensitive strains (MHOM/CN/IN/80/DD8 and MHOH/CN/90/9044) of *L. donovani* have been preserved and passaged in the golden hamsters. Livers and spleens of sick golden hamsters were homogenized and maintained in M199 medium (Sigma-Aldrich St. Louis, MO, USA) supplemented with 10% fetal bovine serum (FBS; Gibco, Franklin, TN, USA) and antibiotics (100 U/ml penicillin and 100 μg/ml streptomycin; Hyclone, Logan, UT, USA) at pH 7.4 and 28 °C for 5–7 days to culture promastigotes.

Sodium stibogluconate (SSG; MCE, Monmouth Junction, NJ, USA) and potassium antimonyl tartrate trihydrate (PAT; Sigma-Aldrich St. Louis, MO, USA) were dissolved in ultrapure water for a stock concentration of 10 mM. A murine macrophage stable cell line RAW 264.7 (Jennio, Guangzhou, China) was recovered and maintained according to the manufacturer’s instructions. Golden hamsters (Mesocricetus auratus, 8–10 weeks-old, female) were purchased from the Chengdu Institute of Biological Products Co., Ltd, Chengdu, China. Female wild-type BALB/c mice aged 6–7 weeks were purchased from Dassy (Experimental animals Co., Ltd, Chengdu, China), and maintained under standard conditions.

### *In vitro* promastigote culture

To test the likely antimony-resistance of the clinical isolate (HCZ) of *L. donovani* [18], logarithmic phase promastigotes of HCZ isolate and reference sensitive strains DD8 and 9044 were harvested, washed, and cultured in M199 medium (Sigma-Aldrich) supplemented with 10% FBS (Gibco), antibiotics (100 U/ml penicillin and 100 μg/ml streptomycin) and 65 μM of PAT (cf. [32]), at a cell density of 1.0 × 10^6^ cells/well. Same volume of phosphate-buffered saline (PBS) was used as control. Following 24 and 48 h of incubation with PAT, the survival rate of promastigotes of *L. donovani* strains tested was determined by a flow cytometry analysis. In brief, promastigote samples of HCZ isolate, DD8 and 9044 strains were collected by centrifuge at 800× *g* and purified by three washes in PBS at 4 °C; purified promastigotes were then processed using a FITC Annexin V Apoptosis Detection Kit I (BD, Franklin Lakes, NJ, USA) according to the manufacturer’s instructions, then analysed by a flow cytometry machine (CytoFLEX LX; BD). Inhibition rate (%) = 100% − [(No. of live promastigotes in treated sample/ No. of live promastigotes in control) × 100%].

### Macrophage infection

RAW 264.7 cells (~ 5.0 × 10^5^ cells/per well) were plated on round glass coverslips in 24-well plates and allowed to adhere to the slides for 24 h at 37 °C, 5% CO_2_ with RPMI-1640 medium (Sigma-Aldrich) supplemented with 10% FBS (Gibco). Stationary phase promastigotes (~ 5.0 × 10^6^ cells/per well) of HCZ isolate, DD8 and 9044 strains were added into wells, and incubated at 37 °C, 5% CO_2_ for 6 h. Next, the non-infected promastigotes were removed, and the infected macrophages were incubated at 37 °C in 5% CO_2_ with fresh medium without drugs for 24 h. The medium was then removed, and fresh culture medium supplemented with 10 μM SSG (cf. [33]) were added into wells for 2 days, with PBS used as control. At 24 and 48 h, the glass coverslips were fixed in methanol (Solarbio, Beijing, China) and stained with Wright’s stain (Solarbio) to calculate the number of intracellular amastigotes under light microscopy by counting 200 macrophages per slide. Inhibition rate (%) = 100% − [(No. of amastigotes in treated sample/ No. of amastigotes in control) × 100%].

### Animal infection and treatment with SSG

All experimental mice under a standard condition were randomly divided into three groups, with 12 mice included in each group. Stationary phase promastigotes, collected from the culture medium, were centrifuged at 1000× *g* for 10 min, resuspend in PBS. Using an intravenous approach, promastigotes (~ 1.0 × 10^7^ cells) of HCZ isolate, DD8 and 9044 strains of *L. donovani* were inoculated in three groups of mice, respectively. BALB/c mice of three groups were treated with SSG at a single dose of 70 mg/kg (cf. [34]) body weight of the animal or PBS *via* intraperitoneal injection at 14 days after infection. All mice were euthanized and dissected after 14 days of treatment. The livers and spleens were harvested, weighed, and smears (Wright’s staining) were prepared and examined by light microscopy. The parasite burdens were indicated as Leishman-Donovan Units (LDU) = No. of amastigotes per 1000 nucleated cells × liver or spleen weight (g).

### DNA extraction and sequencing

Genomic DNA were isolated from promastigotes representing each of the HCZ isolate, DD8 and 9044 strains of *L. donovani* using TIANamp Genomic DNA Kit (Tiangen, Beijing, China) according to the manufacturer’s instructions. In brief, purified stationary phase promastigotes (~ 4 × 10^8^ cells) were transferred to a sterile microcentrifuge tube containing lysis buffer, from which the released genomic DNA was bound to column, washed with washing buffer, and then eluted with nuclease-free water. DNA samples representing the HCZ isolate, DD8 and 9044 strains were stored at -20 °C until use.

The quality and intensity of DNA samples were measured using a Qubit Fluorometer (Thermo Fisher Scientific, Rochester, NY, USA). Sequencing libraries (pair-end) representing each *L. donovani* samples (HCZ isoalte, DD8 and 9044 strains) were constructed using an internal DNA 200 bp-800 bp Insert Size Pooling Library Construction Kit (BGI, Shengzheng, China). In brief, 1 µg genomic DNA was randomly fragmented, incubated with End Repair Mix, combined with A-Tailing Mix, ligated to Illumina adapters, amplified with PCR Primer Cocktail and PCR Master Mix, and validated by the Agilent 2100 Bioanalyzer and ABI StepOnePlus Real-Time PCR System. The qualified libraries were sequenced on Illumina Hiseq4000 (PE150) platform.

### Genomic mutation analysis

Pair-end raw reads were processed to remove sequencing adaptors and low-quality reads for quality control. Processed reads were mapped to the reference genome of *L. donovani* (BPK282A1 strain; BioProject: PRJEA61817) using the BWA software v.0.7.15 [35]. Following genome mapping, contaminated reads were removed while mapped reads were summarised for statistical analyses of mapping rate, sequencing depth and genomic coverage for HCZ isolate, DD8 and 9044 strains of each *L. donovani* strain. Structural variation (SV), copy number variation (CNV), single nucleotide polymorphism (SNP) and insertion/deletion (indel) were detected by comparing mapped reads with the reference genome using BreakDancer v.1.1 [36], CNVnator v.0.3 [37] and GATK v.4.0 [38]. Detected SNPs were filtered using parameters ExcessHet > 54.69, QD < 2.0, MQ < 40.0, FS > 60.0, SOR > 3.0, MQRankSum < -12.5 and ReadPosRankSum < -8.0. Identified Indels were checked based on thresholds ExcessHet > 54.69, QD < 2.0, FS > 200.0, SOR > 10.0, MQRankSum < -12.5 and ReadPosRankSum < -8.0. Filtered SNPs and Indels were annotated using ANNOVAR software [39].

### Protein isolation and LC-MS/MS

Proteins were extracted from the same batch of *L. donovani* samples used for the genomic DNA isolation. In brief, the purified promastigotes were incubated in lysis buffer (8 M urea and 1% protease inhibitor cocktail) at 4 °C for 3 min, sonicated on ice for three times using a high intensity ultrasonic processor (Scientz, Ningbo, China). Sonicated samples were centrifuged at 12,000× *g* at 4°C for 10 min to remove debris. Supernatant was collected, and the protein concentration was measured using a BCA Protein Assay Kit (Thermo Fisher Scientific).

For each of the *L. donovani* samples (HCZ isolate, DD8 and 9044 strains), proteins were subjected to an in-solution reduction, alkylation and digestion approach. In brief, 300 µg protein aliquots (*n* = 3) was reduced with 5 mM dithiothreitol at 56 °C for 30 min, alkylated with 11 mM iodoacetamide at room temperature in darkness for 15 min, digested with trypsin (1:50 trypsin-to-protein) at 37 °C overnight, and further digested with trypsin (1:100 trypsin-to-protein) at 37 °C for 4 h. The processed samples were dissolved in 1.0% (v/v) formic acid, and then subjected to liquid chromatography–mass spectrometry/mass spectrometry (LC-MS/MS) analysis using a Q ExactiveTM Plus Orbitrap mass spectrometer (Thermo Fisher Scientific) coupled online to the EASY-nLC 1000 UPLC system.

### Proteomic data analysis

Mass spectrometry data were processed using MaxQuant search engine v.1.5.2.8 [40]. In brief, tandem mass spectra were search against the reference proteome of *L. donovani* (BPK282A1 strain; BioProject: PRJEA61817) to identify and quantify peptides. Trypsin/P was specified as cleavage enzyme (≤ 4 missing cleavages), mass tolerances for precursor ions were set as 20 ppm for the first search and 5 ppm for the main search, and mass tolerance for fragment ions as 0.02 Da. False discovery rate (FDR) was adjusted to < 1% and minimum score for modified peptides was set > 40. Gene Ontology (GO), domain architecture, Kyoto Encyclopedia of Genes and Genomes (KEGG) and subcellular localisation annotations were performed for the identified proteins based on UniProt-GOA database, InterPro, KEGG database and Wolfpsort databases using an internal R script.

### Comparative and integrative analyses

Pairwise comparisons were conducted among HCZ isolate, DD8 and 9044 strains of *L. donovani* at genomic and proteomic levels. Genomic mutations (e.g. CNV, SNV and frameshift for protein-coding genes) and protein abundances (i.e. qualitative and quantitative data sets) were compared between HCZ and DD8, HCZ and 9044, DD8 and 9044 to infer specific genomic mutations and protein expression patterns in HCZ isolate of *L. donovani*. Perseus software [41, 42] was used to analyse and visualise proteomic datasets. Principal components analysis (PCA) and hierarchical cluster analysis (HCA) were performed to analyse differences among the proteomic datasets representing distinct strains of *L. donovani*. Venn diagram was drawn using a R package Venn Diagram v.1.6.20. Cross-comparison of genomic and proteomic data was performed for an integrative analysis of the relationship between mutations at DNA level and expression changes at protein level.

### Statistical analysis

All experiments were repeated a minimum of three separate times. All data analyses and graphs were performed with GraphPad Prism 6.0 software (San Diego, CA, USA). The data were analyzed using Student’s t-test for comparison of two groups and were expressed as the means ± standard deviation (SD). Significant differences were determined and designated with asterisks as follows: *P < 0.05; **P < 0.01; ***P < 0.001 and ****P < 0.0001.

## Results

### *Leishmania donovani* HCZ isolate was antimony-resistant in promastigote culture and in infected macrophages *in vitro*

**Fig. 1:**
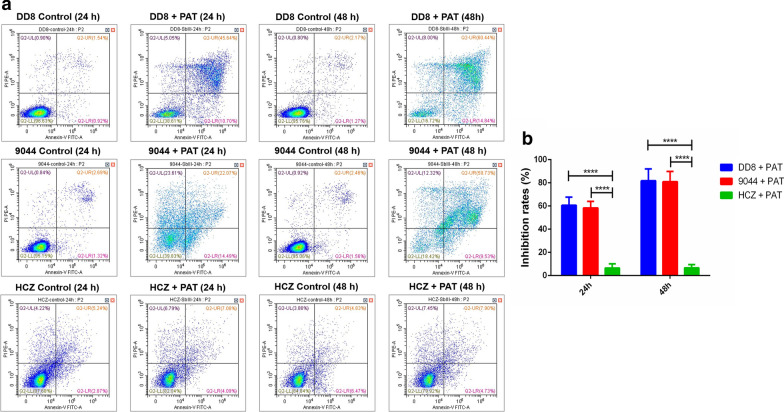
Flow cytometry analysis of *Leishmania donovani* promastigotes exposed to PAT and inhibition rates of PAT on promastigotes. **a** Survival rates of promastigotes representing reference sensitive strains (9044 and DD8), and clinical isolate (HCZ) of *L*. *donovani* after 24 and 48 h of incubation with PBS (control) and PAT are indicated in the lower left quadrant (LL quadrant). **b** Inhibition rates of PAT on the survival of promastigotes of DD8 and 9044 strains and HCZ isolate after 24 and 48 h of incubation. All experiments were carried out in triplicates and figures shown are representative of these experiments. ****P < 0.0001

**Fig. 2:**
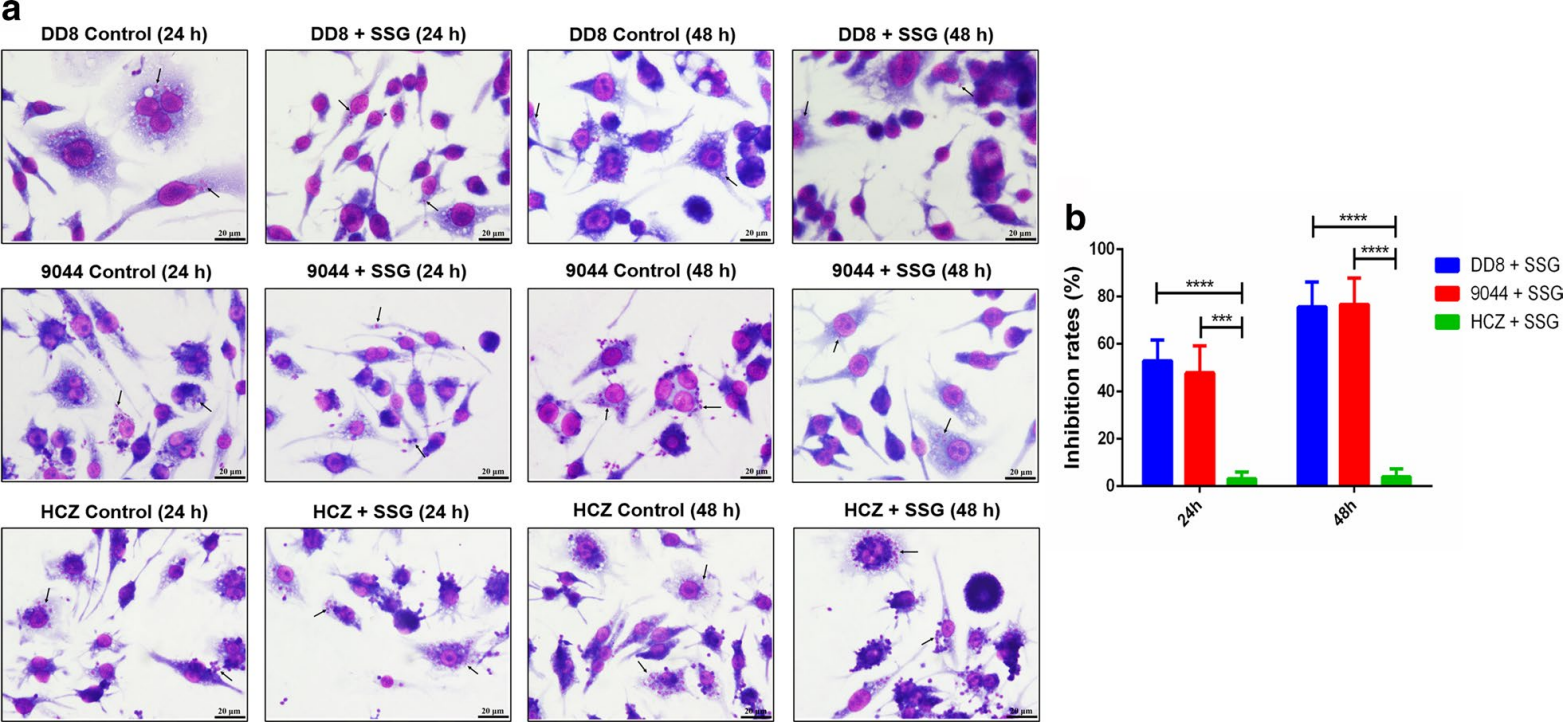
Wright’s staining of intracellular amastigotes in cells after treated with SSG and inhibition rates of SSG on intracellular amastigotes. **a** Intracellular amastigotes representing reference sensitive 9044 and DD8 strains, and HCZ isolate of *L*. *donovani* after 24 and 48 h of incubation with PBS (control) and SSG. **b** Inhibition rates of SSG on the number of intracellular amastigotes in macrophages after 24 and 48 h of treatment. At least three independent experiments were carried out and figures shown are representative of these experiments. The black arrows indicated intracellular amastigotes. ***P < 0.001, ****P < 0.0001. *Scale-bars*: 20 µm

Following incubation with PAT *in vitro*, flow cytometry analysis revealed similar survival rates of promastigotes between DD8 and 9044 strains, but different survival rates between HCZ isolate and DD8 strain, and between HCZ isolate and 9044 strain of *L. donovani*. Specifically, compared with the low survival rates of DD8 (38.61% and 16.72%) and 9044 strains (39.83% and 18.42%) of *L. donovani* after 24 and 48 h of incubation with PAT (65 µM), HCZ isolate appeared to be more robust (82.04% and 79.92%) (Fig. 1a). In addition, lower number of intracellular amastigotes were observed in the macrophages infected with 9044 and DD8 strains after treatment (SSG, 10 µM), whereas more amastigotes were found in HCZ isolate infected macrophages (Fig. 2a). For HCZ isolate, the inhibitory effects of PAT on promastigotes (HCZ *vs* DD8: *t*_(8)_ = 10.0549, *P* < 0.0001, 24 h; *t*_(8)_ = 13.9502, *P* < 0.0001, 48 h) (HCZ *vs* 9044: *t*_(8)_ = 11.0015, *P* < 0.0001, 24 h; *t*_(8)_ = 15.7577, *P* < 0.0001, 48 h) *in vitro* and SSG on intracellular amastigotes in macrophages (HCZ *vs* DD8: *t*_(8)_ = 8.4122, *P* < 0.0001, 24 h; *t*_(8)_ = 12.105, *P* < 0.0001, 48 h) (HCZ *vs* 9044: *t*_(8)_ = 6.5992, *P* = 0.0002, 24 h; *t*_(8)_ = 10.7526, *P* < 0.0001, 48 h) were significantly weaker than that for 9044 and DD8 strains (Figs. 1b and 2b), indicating antimony-resistance of *L. donovani* HCZ strain *in vitro*.

### *Leishmania donovani* HCZ isolate showed strong virulence and resistance *in vivo*

**Fig. 3:**
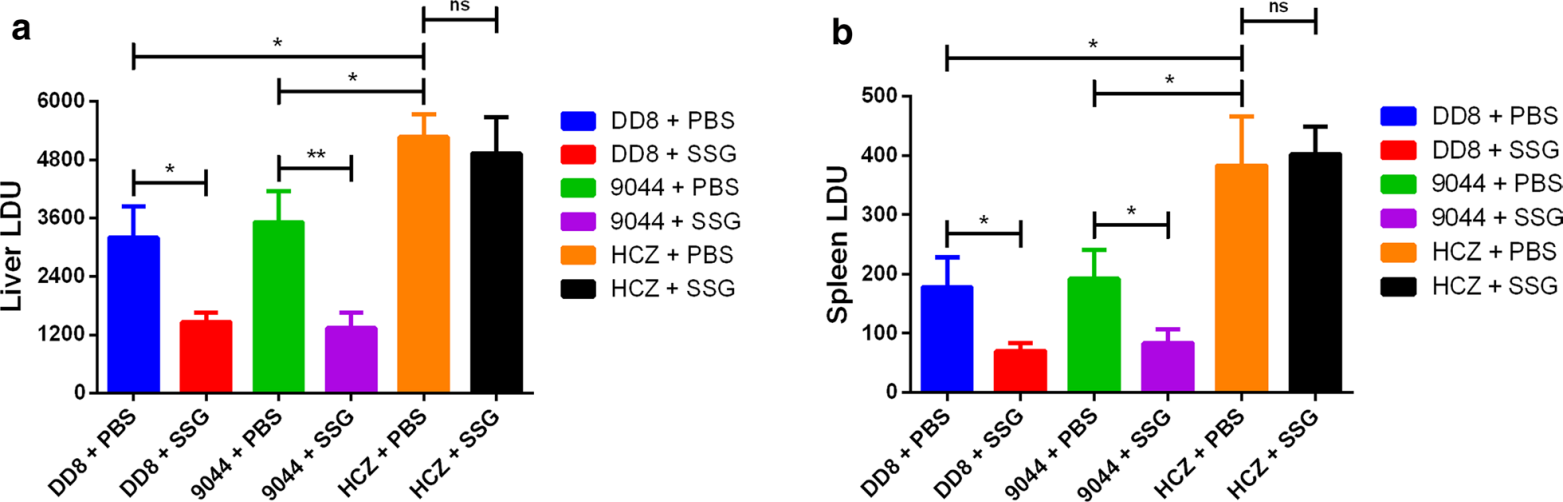
Parasite loads during *Leishmania donovani* infection after treatment with SSG or PBS. Liver (**a**) and spleen (**b**) parasite loads in SSG- or PBS-treated *L. donovani* (DD8, 9044 and HCZ strains) infected BALB/c mice. Parasite burdens in spleen and liver were expressed as LDU ± SD of the mean. The data are mean values from three independent experiments. *P < 0.05, **P < 0.01, ns: no significance

To further confirm the antimony-resistant phenotype of HCZ isolate of *L. donovani*, resistance testing was conducted in a mice infection model. By comparing the parasite loads of livers and spleens of BABL/c mice in SSG-treated and PBS-treated groups, HCZ isolate appeared to be more pathogenic and more resistant to antimony than DD8 and 9044 strains *in vivo* (Fig. 3).

Specifically, although parasite loads were observed in all mice infected with DD8 and 9044 strains, and HCZ isolate of *L. donovani*, the LDU of liver in the latter group was significantly higher than other two groups (HCZ + PBS *vs* DD8 + PBS: *t*_(4)_ = 4.5104, *P* = 0.0107; HCZ + PBS *vs* 9044 + PBS: *t*_(4)_ = 3.838, *P* = 0.0185) (Fig. 3a). Similar to the LDU of liver, the LDU of spleen in HCZ + PBS group was significantly higher than that in other two groups (HCZ + PBS *vs* DD8 + PBS: *t*_(4)_ = 3.6541, *P* = 0.0217; HCZ + PBS *vs* 9044 + PBS: *t*_(4)_ = 3.4466, *P* = 0.0261) (Fig. 3b). Additionally, SSG treatment efficiently eliminated the intracellular amastigotes of 9044 and DD8 strains, but not HCZ isolate. Specifically, the LDU of liver (DD8 + SSG *vs* DD8 + PBS: *t*_(4)_ = 4.4701, *P* = 0.0111; 9044 + SSG *vs* 9044 + PBS: *t*_(4)_ = 5.3143, *P* = 0.006) and spleen (DD8 + SSG *vs* DD8 + PBS: *t*_(4)_ = 3.5172, *P* = 0.0245; 9044 + SSG *vs* 9044 + PBS: *t*_(4)_ = 3.496, *P* = 0.025) in DD8 and 9044 groups significantly decreased. On the contrary, the LDU of liver (HCZ + SSG *vs* HCZ + PBS: *t*_(4)_ = 0.6506, *P* = 0.5508) and spleen (HCZ + SSG *vs* HCZ + PBS: *t*_(4)_ = 0.3546, *P* = 0.7408) in HCZ infected BABL/c mice did not significantly reduce after treatment of SSG, showing clear antimony-resistance of *L. donovani* HCZ isolate *in vivo* (Fig. 3).

### Genomic mutations identified in *L. donovani* HCZ isolate, 9044 and DD8 strains

More than 7.80 million raw reads were produced for each *L. donovani* isolate or strain (HCZ, 9044 and DD8) (Additional file 1: Table S1). By aligning the raw reads to the reference genome of this parasite (BPK282A1 strain; BioProject: PRJEA61817), at least 4.79 million reads were mapped for each of the three *L. donovani* strains (Table 1). Specifically, mapped reads for the drug-resistant clinical isolate (HCZ) of *L. donovani* covered > 90% of the reference genome (BPK282A1 strain) at a ≥ 10-fold coverage (Table 1). Similar genome coverages (94.79% and 94.66%) were also achieved for the drug-susceptible strains (9044 and DD8) of this parasite (Table 1).

**Table 1:** Statistical summary of genome resequencing and genomic analyses of *L. donovani* strains

Strain	Mapped reads	Coverage 10× (%)	CNVs	5’UTRs	Frameshifts	Stop-gain	Stop-loss
HCZ	4,791,654	90.54	517	3	549	56	22
9044	10,197,272	94.79	670	2	431	59	22
DD8	10,051,566	94.66	715	0	122	9	3

By comparing the mapped reads with the reference genome (BPK282A1 strain), hundreds of structural variations including 517, 670 and 715 copy number variations (CNVs) were identified in *L. donovani* HCZ isolate, 9044 and DD8 strains studied, respectively (Table 1; Additional file 1: Table S1). Comparisons with the reference genome (BPK282A1 strain) also revealed thousands of single nucleotide polymorphisms (SNPs) and single nucleotide variations (SNV; i.e. insertions and deletions) in the genomes of the HCZ isolate, 9044 and DD8 strains (Additional file 1: Table S1). Specifically, compared with the reference strain, > 130,000 SNPs were identified in *L. donovani* HCZ isolate, including 50,680 sites in protein-coding regions, 23 in non-coding RNA (ncRNA)-coding regions, and three in 5′ untranslated regions (5′UTRs). These variations resulted in numbers of synonymous and nonsynonymous mutations in protein-coding and non-coding genes, such as frameshift (*n* = 549, 431 and 122), stop-gain (*n* = 56, 59 and 9) and stop-loss (*n* = 22, 22 and 3) mutations in the *L. donovani* HCZ, 9044 and DD8 strains, respectively (Table 1; Additional file 1: Table S1).

### Specific genomic mutations identified in the resistant clinical isolate (HCZ) of *L. donovani*

**Fig. 4:**
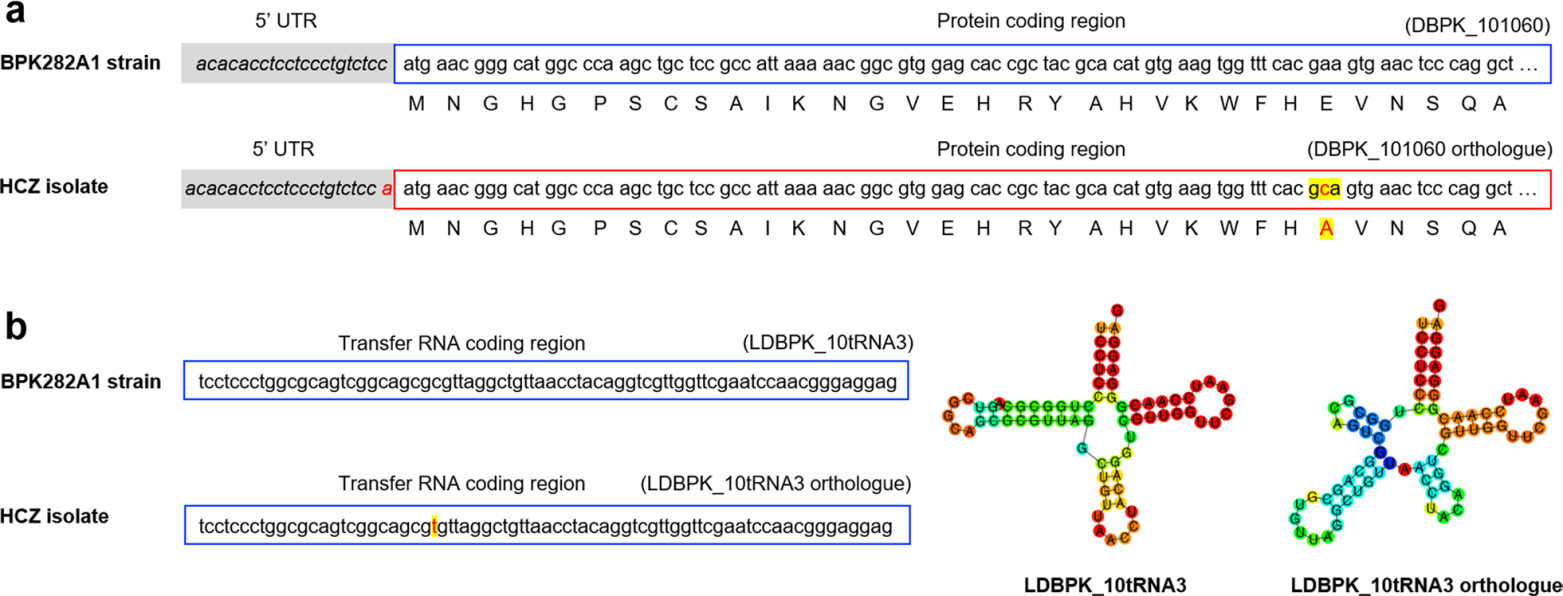
Example of genomic mutations identified in the drug-resistant clinical isolate of *Leishmania donovani*. **a** An insertion mutation of adenine in 5’ untranslated region (UTR) and a base alteration (from adenine to cytosine) in protein-coding region are identified in the DBPK_101060 (HCZ) isolate of *L. donovani*, compared with reference BPK282A1 strain. These mutations are inferred to link to changes in messenger RNA transcription, protein expression and molecular functions. **b** A site mutation (from cytosine to thymine) is detected in a transfer RNA (tRNA) coding region, which might change the secondary structure of this non-coding RNA

Genetic mutations exclusively occurred in the clinical isolate of *L. donovani* might contribute to fitness-gains allowing for virulence and resistance. By pairwise comparisons (DD8 *vs* 9044; DD8 *vs* HCZ; 9044 *vs* HCZ), genetic variations in HCZ isolate but not in DD8 and 9044 strains were identified, including 86 copy number variations and 271 frameshift mutations (e.g. stop-gain and stop-loss mutations) in protein-coding genes, and two site mutations in non-coding genes (Additional file 1: Tables S2-S5). Specifically, one site mutation (adenine to cytosine) in the coding region and one insert mutation in the 5’ UTR were identified for a histone H3 protein-coding gene homologue (locus tag: DBPK_101060) (Fig. 4a). In addition, a site mutation was detected in the coding region of a transfer RNA (LDBPK_10tRNA3) coding gene orthologue, which was inferred to change the secondary structure of this molecule (Fig. 4b).

### Proteomes of *L. donovani* HCZ, 9044 and DD8 strains

**Fig. 5:**
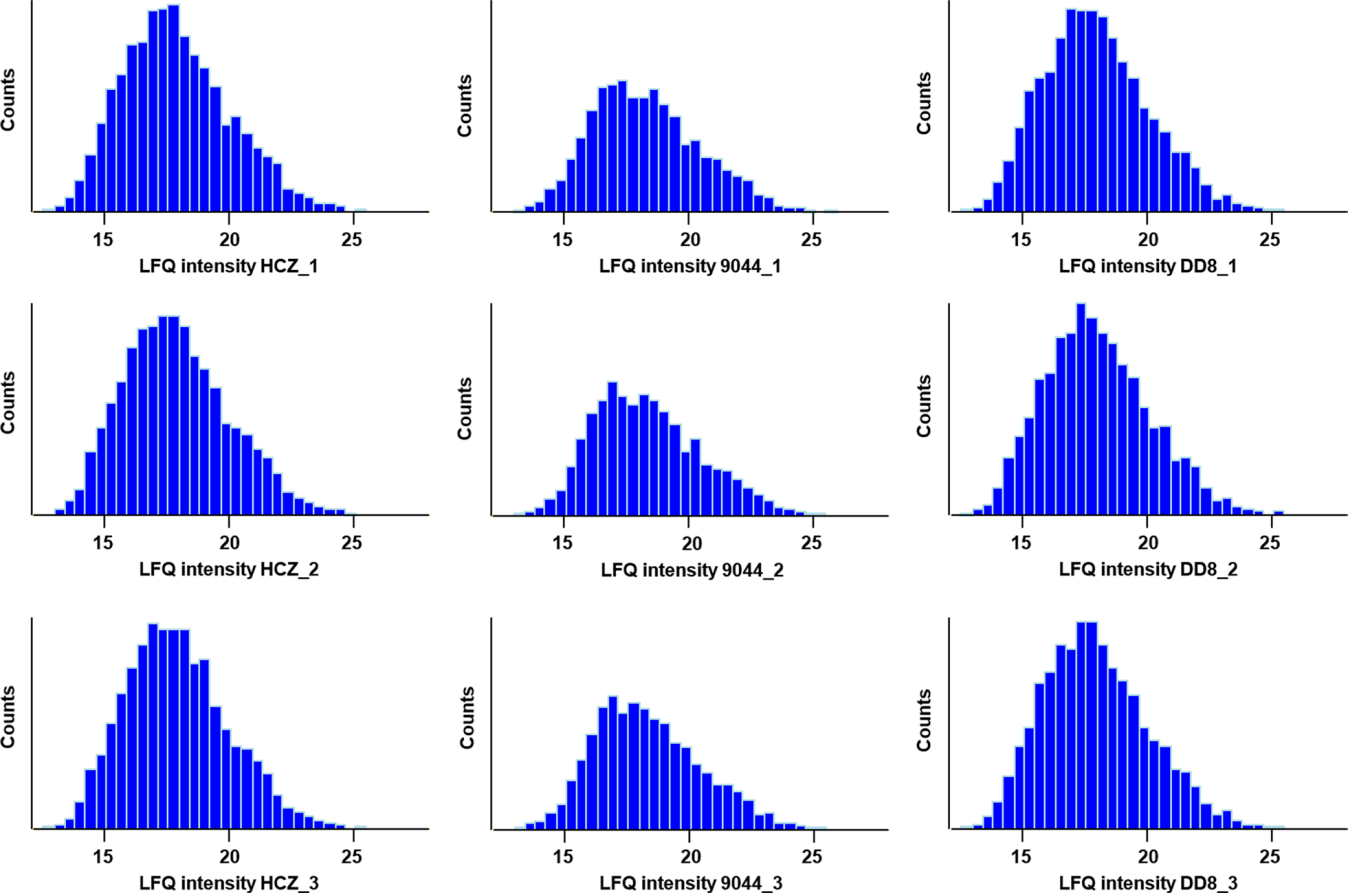
Histogram of protein abundances detected in HCZ, DD8 and 9044 strains of *Leishmania donovani*. Diagrams indicating the gaussian distribution of protein intensity measured in replicated samples (*n* = 3) of the resistant clinical isolate (HCZ) and the susceptible strains (9044 and DD8) of *L. donovani*

To verify the genetic mutations in protein-coding genes and explore its biological influence at post-genomic level, proteomic analysis was conducted for *L. donovani* HCZ, DD8 and 9044 strains. In total, 544,057 secondary spectrums were detected from the three strains, 86.5% of which were matched to the reference proteome database for *L. donovani* (BPK282A1 strain; BioProject: PRJEA61817) (Table 2). From these mapped spectrums, 73,792 peptides were identified, representing 7412 proteins/protein groups of *L. donovani* strains analysed (Table 2; Additional file 1: Table S6). Based on unique peptides detected (*n* = 35,752, 15,761 and 36,190), 7287, 7267 and 6489 proteins were identified in HCZ, 9044 and DD8 strains of *L. donovani*, respectively. Most of these proteins were detected with ≥ 2 unique peptides, and the number of proteins that could be quantified (*n* = 6122, 5918 and 3962) varied among the three strains of *L. donovani* (Table 2, Fig. 5). Although similar number of proteins were detected in the resistant HCZ isolate and susceptible 9044 strain (Table 2), molecules identified in the latter appeared to be expressed with lower intensities (Fig. 5), suggesting potential molecular regulations at post-genomic level.

**Table 2:** Statistical summary of proteomic analyses of *L. donovani* strains

Strain	Total spectrum	Matched spectrum (%)	Total peptides	Total proteins	Unique peptides	Identified proteins	Quantified proteins
HCZ	544,057	470,565 (86.5%)	73,792	7412	35,752	7287	6122
9044					15,761	7267	5918
DD8					36,190	6489	3962

### Specific protein expression changes in the resistant clinical isolate of *L. donovani*

**Fig. 6:**
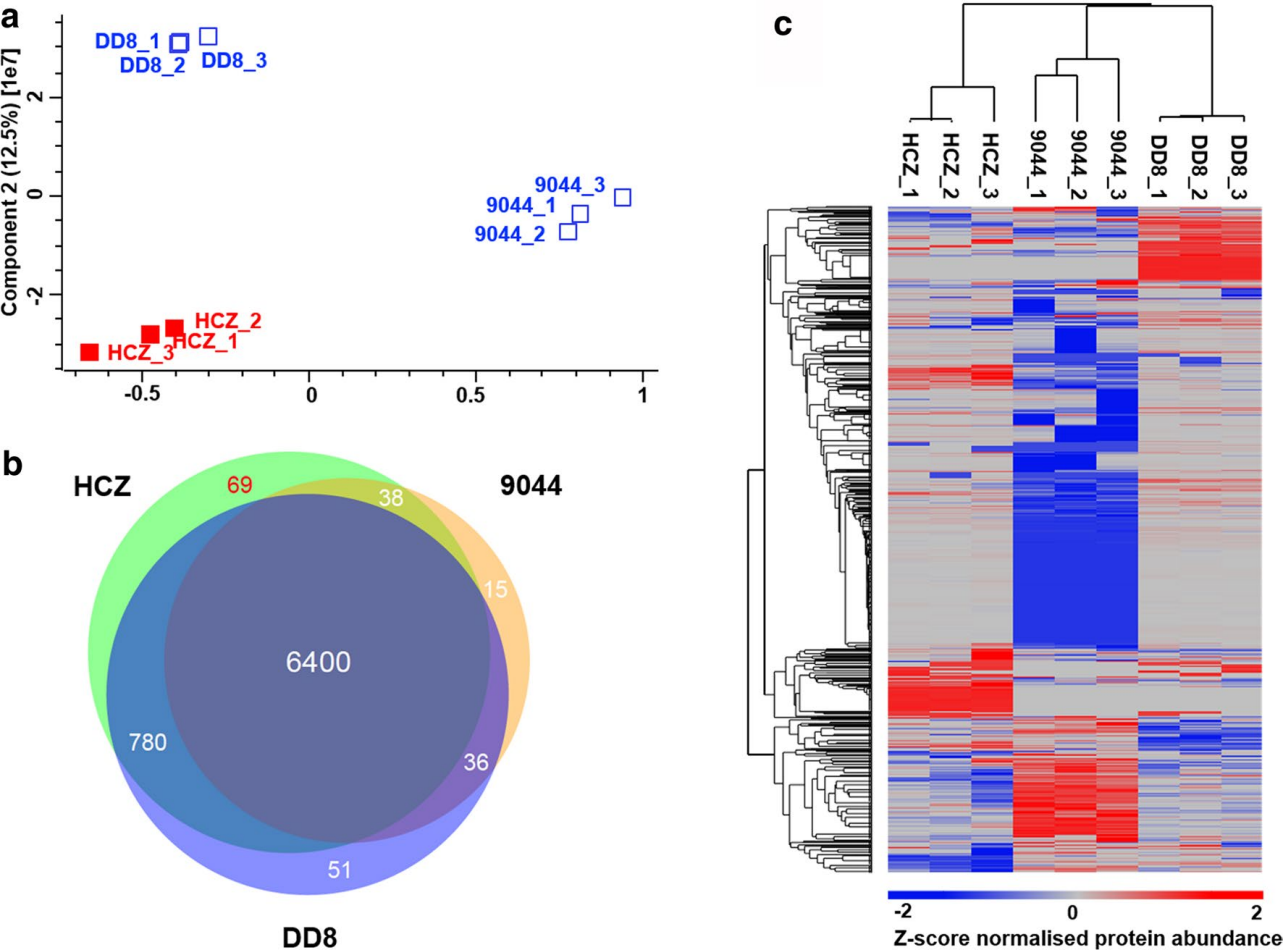
Comparative analysis of protein expression among HCZ, DD8 and 9044 strains of *Leishmania donovani*. **a** Principal components analysis (PCA) of proteomic datasets of *L. donovani* HCZ, DD8 and 9044 strains. **b** Venn diagram showing the identification of proteins in HCZ, DD8 and 9044 strains of *L. donovani*. **c** Hierarchical cluster analysis (HCA) indicating the quantification of proteins in HCZ, DD8 and 9044 strains of *L. donovani*

Different profiles of protein expression were detected among *L. donovani* strains studied in this study (Fig. 6). A principal components analysis of proteomic data sets showed three distinct clusters representing HCZ, 9044 and DD8 strains of *L. donovani* (Fig. 6a), suggesting important biological differences between the drug-resistant and drug-susceptible strains of this parasite. Pairwise comparisons of these proteomic data sets revealed that 6400 proteins (86.34%) were detected in all strains, and some molecules were exclusively identified in HCZ, 9044 or DD8 strain (Fig. 6b, Additional file 1: Table S7). Specifically, 69 proteins were only detected in the resistant clinical isolate (HCZ) of *L. donovani*, 51 proteins in DD8 strain, and 15 proteins in 9044 strain of this parasite (Fig 6b, Additional file 1: Table S7). Particularly, a hierarchical clustering of quantitative proteomic datasets unveiled different regulations of protein expression among HCZ, 9044 and DD8 strains of *L. donovani* (Fig. 6c). Such significant discrepancies among the three strains of *L. donovani* indicated differences in protein expression between the sensitive and resistant strains of this parasite.

**Fig. 7:**
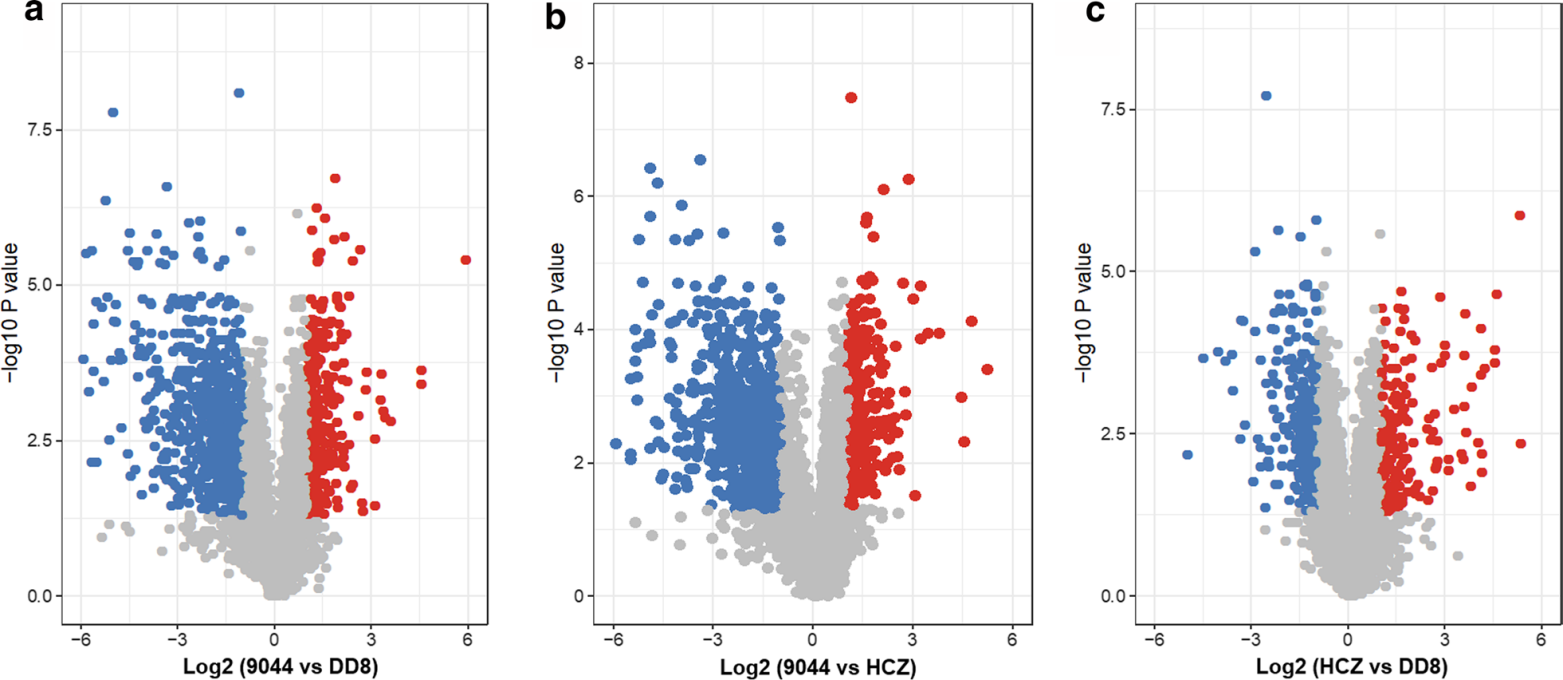
Pairwise comparisons of protein expression among DD8 and 9044 strains and HCZ isolate of *Leishmania donovani*. Differential protein expression analyses between (**a**) DD8 and 9044 strains, (**b**) HCZ isolate and 9044 strain, and (**c**) HCZ isolate and DD8 strain of *L. donovani*. Proteins detected with significantly higher, lower and unchanged abundances in each comparison group are indicated in red, blue and grey, respectively

Significant differences (FC ≥ 2 and *P* < 0.05) in protein expression were identified between DD8 and 9044 (*n* = 973), 9044 and HCZ (*n* = 967), as well as DD8 and HCZ (*n* = 433) of *L. donovani* (Fig. 7, Additional file 1: Table S8). By removing the natural variations indicated between DD8 and 9044 strains, the number of proteins differentially expressed between resistant HCZ isolate and reference sensitive DD8/9044 strain reduced to 100, including 59 upregulated proteins (e.g. cysteine peptidase, paraflagellar rod component, dehydrogenases, glutathione S-transferase, inosine-adenosine-guanosine-nucleosidehydrolase, NADH-ubiquinone oxidoreductase, nitrate reductase, phospholipid-transporting ATPase, tubulin beta chain, kinesin), 23 down-regulated molecules (e.g. ABC-thiol transporter, gamma-glutamylcysteine synthetase, glucosamine-fructose-6-phosphate aminotransferase, fatty acyl CoA syntetase 1), and 18 proteins with inconsistency (Additional file 1: Table S8).

### Integrated genomic mutations and protein expression changes in *L. donovani* HCZ isolate

**Fig. 8:**
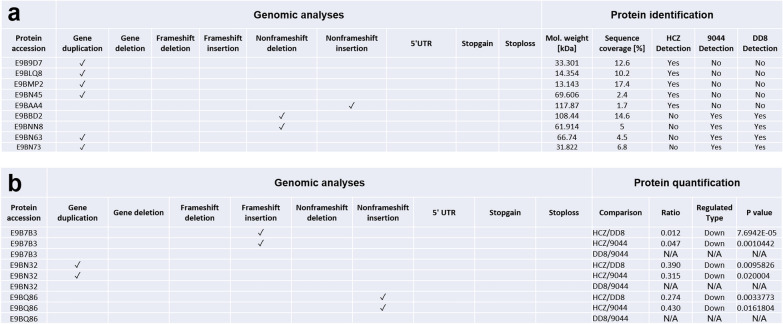
Integration of genomic mutations and protein expression changes in HCZ, 9044 and DD8 strains of *Leishmania donovani*. **a** Integration of gene duplication, gene deletion, frameshift deletion, frameshift insertion, non-frameshift deletion, non-frameshift insertion, mutations in 5’ untranslated region (5’ UTR), stop-gain, stop-loss and exclusive protein detection among *L. donovani* HCZ, 9044 and DD8. **b** Integration of gene duplication, gene deletion, frameshift deletion, frameshift insertion, non-frameshift deletion, non-frameshift insertion, mutations in 5’ untranslated region (5’ UTR), stop-gain, stop-loss and exclusive protein expression changes (up-/down-regulation) between HCZ and 9044, and between HCZ and DD8 strains of *L. donovani*

By integrating genomic and proteomic datasets, relationships between the genomic mutations identified in protein-coding genes and the abundances of detected proteins were established (Additional file 1: Tables S9 and S10). Predominant genomic mutations linked to significant changes in protein abundance were gene deletion, gene duplication, non-frameshift deletion and non-frameshift insertion. Specifically, gene duplication mutations resulted in exclusive protein expression (E9B9D7, E9BLQ8, E9BMP2 and E9BN45) in the drug-resistant clinical isolate (HCZ) (Fig. 8a, Additional file 1: Table S9). In addition, gene duplication, frameshift insertion and non-frameshift insertion linked to significant downregulated (FC ≥ 2 and *P* < 0.05) abundances of three proteins (E9BN32, E9B7B3 and E9BQ86) in the resistant HCZ isolate (Fig. 8b, Additional file 1: Table S10). Although most of these molecules were uncharacterised proteins, some of them were inferred to be associated with nucleotide-binding, fatty acid metabolism (biosynthesis and degradation), oxidation-reduction and transport of drugs (Additional file 1: Tables S9 and S10).

## Discussion

In this study, we confirmed the virulence and antimony-resistance of a previously reported clinical isolate (HCZ) of *L. donovani* [18], compared genomic and proteomic datasets between this drug-resistant isolate and two drug-susceptible strains of *L. donovani*. By excluding natural variations, gene mutations and proteomic variations were identified in the virulent and resistant HCZ isolate. By integrating the phenotypic, genomic and proteomic results, we identified specific genetic features that might contribute to the pathogenicity and antimony-resistance in HCZ isolate of *L. donovani*.

Long-term environmental adaptations usually serve as the drivers of genetic mutations in organisms. The development of virulence and resistance in *L. donovani* is essentially environmental selections of genomic variations allowing for fitness gain. Identifying such variations at DNA level would be useful to unravel the mechanisms underlying pathogenicity and drug resistance, which can be achieved nowadays by high-throughput next generation sequencing of genomic DNA and comparative analyses between/among strains [43]. For instance, a recent longitudinal genome analyses of 10 clinical isolates of *Leishmania* species have shown that gain and loss of gene copies might be the drivers of strain-specific phenotypic variations [27]. Particularly, sequencing the resistant and susceptible strains of *Leishmania* species has revealed a number of mutations (SNPs and CNVs) that might play roles in the resistance of the parasite [26, 28]. Similarly, in the present study, hundreds of CNVs were identified in the HCZ, DD8 and 9044 strains of *L. donovani*, compared with the reference BPK282A1 strain. However, the considerable overlaps between the resistant (HCZ) isolate and susceptible (DD8 and 9044) strains significantly hindered the interpretation of these findings. Nonetheless, by excluding natural variations indicated between DD8 and 9044 strains, variations specifically identified in the resistant isolate (*n* = 86) are more likely involved in the establishment of resistance and/or virulence. However, linking these genomic variations/mutations to pathogenic or resistant phenotypes is still difficult. The difficulty results from the presence of frequent aneuploidy (the condition of having an abnormal number of chromosomes in a haploid set) in *Leishmania* spp., which means adequate read depth is needed to ensure the accuracy of detected genetic variations, such as the loss and gain of gene copies [41]. In addition, limited information at post-genomic (RNA, protein and metabolite) levels also challenges the biological exploration and understanding of the genetic variations [44].

A proteomic approach was elected to address these issues in the present study. Although RNA sequencing and transcriptomic analysis have been extensively used to explore molecular alterations that might be associated with drug resistance in *Leishmania* species [29], such methodology was not employed in this work. This is because post-transcriptional regulations (*via* microRNAs and other non-coding small RNAs) appear to substantially change the genetic information processing from messenger RNA to protein [45, 46]. Information captured at protein expression level might be more reliable to reflect the biological differences between resistant and susceptible strains of the parasite [47]. Indeed, proteomic studies of *Leishmania* spp. have contributed significantly to understanding the surval, infection and drug resistance of this and related parasite [30, 47]. For instance, by comparing the protein abundance between stains of different phenotypes, molecules such as alpha-tubulin, cyclophilin-A, heat-shock proteins, pteridine reductase, superoxide dismutase and tryparedoxin peroxidase have been linked to the resistance and/or virulence of *Leishmania* species [48, 49]. In this study, significant and specific expression changes were identified for 82 proteins between the resistant and susceptible strains of *L. donovani*. Particularly, the exclusively detected (*n* = 69) and non-detected (*n* = 66) proteins in the resistant (HCZ) isolate of *L. donovani* are very likely to be associated with the establishment of drug resistance. However, although increased tolerance of *L. donovani* to antileishmanial drugs has been linked to reduced drug accumulation, increased infectivity and resistance to oxidative stress [50], little is known about the connections between genetic factors and molecular alterations driving such biological processes.

Integrating the genetic variations revealed by comparative genomics and the molecular changes indicated by comparative proteomics might be useful to answer the biological questions pertaining to the virulence and drug resistance in *Leishmania* species. Specifically, a recent work integrated proteomic and metabolomic datasets, which sufficiently revealed that changes in glycoconjugate production and redox homeostasis might result in reduced virulence of *Leishmania* parasite [31]. Therefore, integrating multiple omics datasets might be an efficient way to link biochemistry and molecular biology to phenotypes (e.g. virulence and resistance) [51]. In the present study, although thousands of mutations/variations were identified in protein-coding genes, and substantial changes were indicated in protein abundances, by in-depth integration only 12 mutations at DNA level were directly linked to protein expression alterations in the resistant clinical isolate of *L. donovani* (Fig. 8). Although the functional network of these 12 genes/proteins is not clear, they are very likely play roles in the pathogenesis and establishment of resistance in host, which might be achieved by altering mRNA transcription and cellular signalling. Nonetheless, although insertion and point mutations of H3 coding gene were detected in HCZ isolate, no significant protein expression change of this gene was identified between the resistant isolate and sensitive strains. Clearly, further detailed and integrative studies should be conducted to elucidate such aspects between resistant and susceptible strains of *Leishmania* spp., preferably at DNA, RNA, protein and metabolites levels. Since it is possible to perform high-throughput CRISPR-Cas9 mediated genome editing in *L. donovani* [52–54], further functional studies of these genes and related gene networks would be also useful for more detailed information about the biological alterations allowing for the drug tolerance. Although the present study focused on the promastigote stage of resistant and susceptible strains of *L. donovani*, the findings are expected in the amastigote stage as well, since similar resistant phenotype was identified in the amastigotes of the parasite. Further explorations on the latter stage would provide more comprehensive information about virulence within host animal and tolerance to drugs *in vivo*.

## Conclusions

In conclusion, high-level pathogenicity and evident antimony-resistance were confirmed for the HCZ isolate of *L. donovani*. Integrative genomic, proteomic and phenotypic studies of the resistant HCZ isolate and reference sensitive strains (DD8 and 9044) of *L. donovani* revealed genetic variations likely associated with nucleotide-binding, fatty acid metabolism, oxidation-reduction and transport of drugs. Further functional studies of such aspects would provide novel insights into our understanding of the mechanisms underlying pathogenesis and drug resistance in *L. donovani* and related species.

## Supplementary information

**Additional file 1: Table S1.** Statistical summary of genomic analyses of *Leishmania donovani* strains. **Table S2.** Exclusive copy number variation identified in *Leishmania donovani* HCZ strain. **Table S3.** Exclusive frameshift mutations identified in *Leishmania donovani* HCZ strain. **Table S4.** Exclusive nonsense mutations identified in *Leishmania donovani* HCZ strain. **Table S5.** Exclusive genomic mutations identified in untranslated region and non-coding RNAs of *Leishmania donovani* HCZ strain. **Table S6.** Statistical summary of proteomic analyses of *Leishmania donovani* strains. **Table S7.** Protein identification in *Leishmania donovani* HCZ, DD8 and 9044 strains. **Table S8.** Differential analysis of protein expression among *Leishmania donovani* HCZ, DD8 and 9044 strains. **Table S9.** Integrative analysis of genetic mutation and protein identification in *Leishmania donovani* HCZ strain. **Table S10.** Integrative analysis of genetic mutation and protein quantification in *Leishmania donovani* HCZ strain.

## Data Availability

The datasets supporting the conclusions of this article are included within the article. Genome sequencing data sets have been submitted to the National Center for Biotechnology Information (NCBI) sequence reads archive (SRA), which can be accessed under accession number PRJNA600762. Proteomic data sets have been submitted to the ProteomeXchange Consortium via the PRIDE partner repository under the accession number PXD017089.

## Abbreviations

VL: visceral leishmaniasis
SSG: sodium stibogluconate
PAT: potassium antimonyl tartrate trihydrate
FBS: fetal bovine serum
PBS: phosphate-buffered saline
LDU: Leishman-Donovan Units
FITC: fluorescein isothiocyanate
SV: structural variation
CNV: copy number variation
SNP: single nucleotide polymorphism
LC-MS/MS: liquid chromatographytandem mass spectrometry
FDR: false discovery rate
GO: gene ontology
KEGG: Kyoto encyclopedia of genes and genomes
PCA: principal components analysis
HCA: hierarchical cluster analysis
SD: standard deviation
